# The complete mitochondrial genome of the bait worm, *Marphysa victori* (Annelida; Polychaeta; Eunicida; Eunicidae)

**DOI:** 10.1128/mra.00635-23

**Published:** 2024-01-11

**Authors:** Seongmin Kim, Hana Kim, Keun-Yong Kim, Jung Soo Heo, Biet Thanh Tran, Byung-Kwon Kim

**Affiliations:** 1 Mud Flat Research Team, Gyeonggi Province Maritime & Fisheries Research Institute, Ansan, South Korea; 2 Department of Biodiversity, National Marine Biodiversity Institute of Korea, Seocheon, South Korea; 3 Department of Genetic Analysis, AquaGenTech Co., Ltd., Busan, South Korea; University of California, Riverside, Riverside, California, USA

**Keywords:** bait worm, *Marphysa victori*, mitochondrial genome, polychaete

## Abstract

The complete mitochondrial genome of *Marphysa victori* Lavesque, Daffe, Bonifácio & Hutchings, 2017, was 15,891 bp in length with a GC content of 41%, comprising 13 protein-coding genes, 2 ribosomal RNA genes, and 22 transfer RNA genes. The maximum-likelihood tree showed the closest relationship between *M. victori* and *M. sanguinea*.

## ANNOUNCEMENT

Members of *Marphysa* (Annelida; Polychaeta; Eunicida; Eunicidae) generally inhabit intertidal shores and are widely used as bait in recreational and commercial fishing (1). A bait worm (1) was recently recorded in Korean coastal water (2). This polychaete plays critical roles in circulating seawater through the sediment and purifying the sediment by its feeding activities in the benthic ecosystem (3).

A specimen of *M. victori* was collected from Geoje Island at the southwestern coast of Korea (34°53′16.4″N, 128°28′0.70″E) and deposited in the Marine Bioresource Collection of Gyeonggi Province Maritime & Fisheries Research Institute, Ansan, Republic of Korea (https://fish.gg.go.kr/, Seongmin Kim, smkim1020@gg.go.kr) under a voucher number, GMFRI-I008. Its genomic DNA was extracted using a phenol-chloroform extraction technique (4). The complete mitochondrial genome (mitogenome) was amplified through overlapping PCR runs using AccuPower ProFi Taq PCR PreMix (Bioneer, Daejeon, South Korea) with the following conditions: one cycle of 95°C for 5 min, 35 cycles of 95°C for 20 s, 60°C for 30 s, 68°C for 2 min; and one cycle of 68°C for 5 min. The PCR products were sequenced by the primer walking method using 24 primers (Table 1) on a 3730xl DNA Analyzer (Applied Biosystems, Foster City, CA, USA). The raw sequences were assembled using Sequencher 5.0 (Gene Codes Corp., Ann Arbor, MI, USA) with default parameters. Ambiguous base pairs were validated and refined manually referring to chromatograms. The mitogenomic sequence was annotated using the MITOS web server (5). Transfer RNA (tRNA) genes were identified by tRNAscan-SE 1.21 (6). A nucleotide sequence matrix including the complete mitogenome sequences of 12 polychaete species retrieved from GenBank (http://www.ncbi.nlm.nih.gov/) and that of *M. victori* in this study were aligned using the Clustal W function in BioEdit 7.2.5 (7) to determine the gene boundaries of protein-coding genes (PCGs) and ribosomal RNA genes. A maximum-likelihood (ML) phylogeny tree based on the first and second codon positions of 13 protein-coding genes of 13 polychaete species (including that of *M. victori*) was constructed using GTR + G + I in MEGA7 (8) with 1,000 bootstraps. The sequence was deposited in the GenBank under the accession number MZ052209.

**TABLE 1:** List of PCR primers used to sequence overlapping fragments of the complete mitogenome of the bait worm, *Marphysa victori*

No.	Primer name	Oligonucleotide (5′ -> 3′)
01	Mvi-MT-00166r	ATGAGGAATGCATGGGCAGT
02	Mvi-MT-00923f	TTATCGCAGTTCCAACAGGA
03	Mvi-MT-01185r	GTGTTATGCCACTTATCAGG
04	Mvi-MT-02490f	CACAGTGGCCATGATCCTAA
05	Mvi-MT-02733r	CGRATTACRTCTCGTCATCA
06	Mvi-MT-04323r	CCTCARAAKCTTATTTGKCC
07	Mvi-MT-04104f	CTTTCAGCTCTGTTGCTCAT
08	Mvi-MT-04949f	GAAGAGCCCTATAGATGACT
09	Mvi-MT-05780f	TAACCCTCTATGCCGACGAA
10	Mvi-MT-06604f	GTCCACTCTTCCACTCTAGT
11	Mvi-MT-07481f	AATACCCTCTTCTAACAAGG
12	Mvi-MT-08332r	GAGAATGGGAGGAATTACAA
13	Mvi-MT-09075r	GCTTTTAGATCGGTTTGTCG
14	Mvi-MT-09824r	ATTGCGGCGTTTAGTAGTTT
15	Mvi-MT-10058f	AAACAAGGATTAGAKACCCT
16	Mvi-MT-10467r	CTATGTTACGACTTATCTCC
17	Mvi-MT-11207f	TGACCGTGCAAAGGTAGCAT
18	Mvi-MT-11508r	TAGTCTGTTATCCCTGCGGT
19	Mvi-MT-11561f	TTGGCACCTCGATGTTGGCTTA
20	Mvi-MT-11640f	TACATGAGCTGAGTTCAGAC
21	Mvi-MT-12516f	CCCATACTCTTCATGTGCTG
22	Mvi-MT-13425f	TGCTCCCACTCATTTCTGAT
23	Mvi-MT-14635r	AATCTGCCATCGTGACTCAT
24	Mvi-MT-15034f	ATTAGAGCCAACGCACCTCT

The complete mitogenome of *M. victori* analyzed in this study was a circular molecule of 15,891 bp in length with a GC content of 41%. It encoded 13 PCGs, 2 ribosomal RNA genes, and 22 transfer RNA genes. Its gene composition was identical to those of typical metazoans. Its gene order was identical to those of two congeneric species, *M. sanguinea* and *M. tamurai*, but different from those of typical polychaetes and even species, *Cryptonome barbada* and *Eurythoe complanata*, in the family Amphinomidae belonging to the same order, Eunicida.

**Fig 1:**
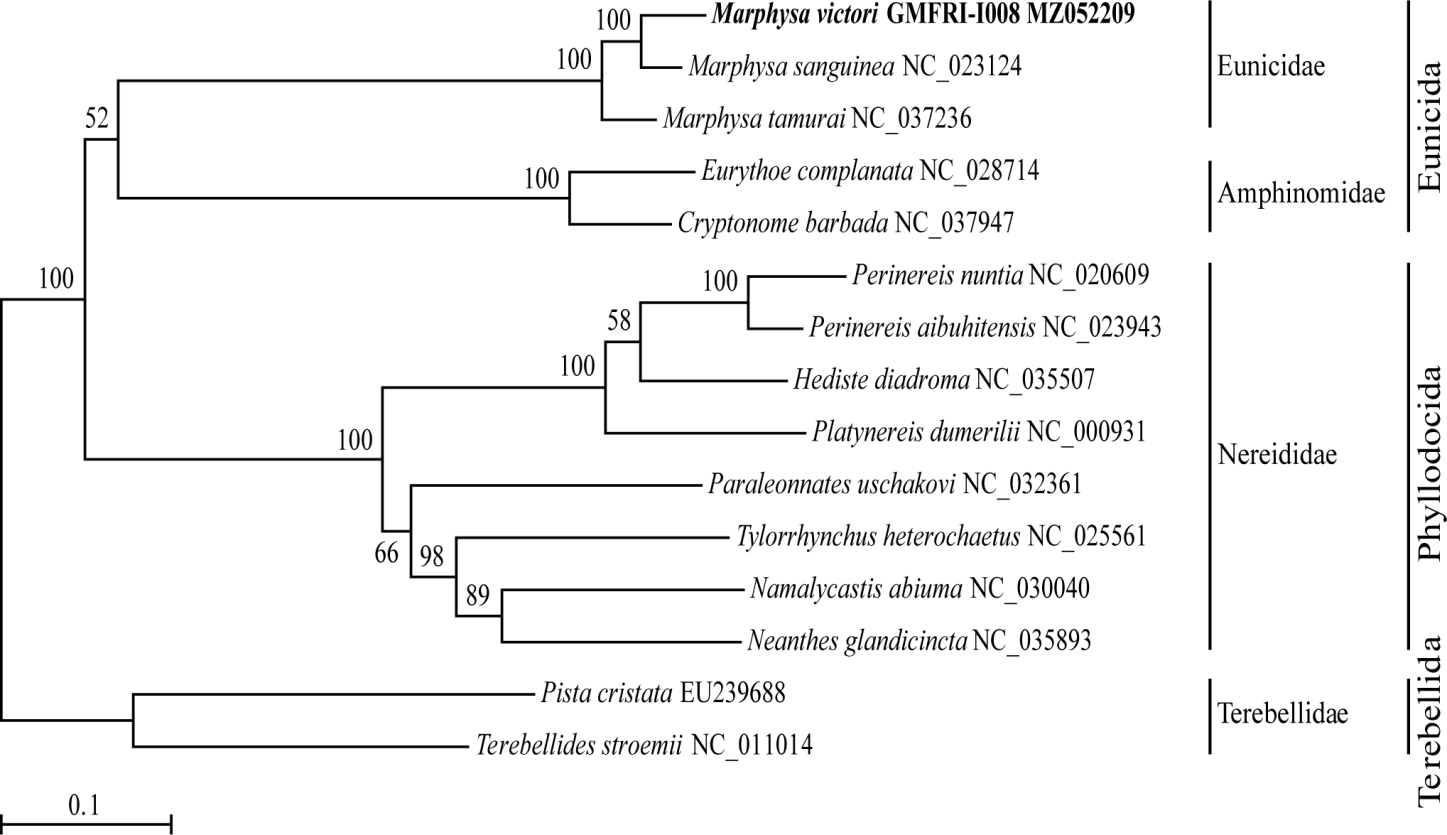
Maximum-likelihood tree based on the complete mitogenome sequences from the species belonging to the order Eunicida (Annelida; Polychaeta). The matrix included the first and second codon positions of 13 protein-coding genes. Bootstrap values above 50% were indicated at each node. The bait worm, *Marphysa victori*, analyzed in this study was shown in bold.

The ML phylogeny tree (Fig. 1) showed that all species belonging to the order Eunicida formed a strongly supported monophyletic group and were separated from species belonging to the order Phyllodocida. All three *Marphysa* species formed a monophyletic group, and *M. victori* in this study showed the closest relationship to *M. sanguinea*.

## Data Availability

The data that support the findings of this study are openly available in GenBank of NCBI under the accession no. MZ052209. Raw reads have been deposited under NCBI BioProject accession number PRJNA989370 and SRA accession numbers from SRX20833117 to SRX20833133.
